# Editorial: Crosstalk between thyroid and heart

**DOI:** 10.3389/fendo.2025.1568023

**Published:** 2025-03-27

**Authors:** Alessandro Pingitore, Iordanis Mourouzis, Fausto Bogazzi

**Affiliations:** ^1^ Clinical Physiology Institute, Council National Research (CNR), Pisa, Italy; ^2^ Department of Pharmacology, Medical School, National and Kapodistrian University of Athens, Athens, Greece; ^3^ School of Medicine, University of Pisa, Pisa, Italy

**Keywords:** thyroid, heart failure, cardiovascular risk, thyroid disfunction, cardioprotection

The intricate interplay between the thyroid and the heart has garnered significant attention due to their essential roles in regulating metabolism and cardiovascular function, respectively. Thyroid hormones profoundly impact cardiac performance, rhythm, and vascular dynamics, while pathophysiological conditions of the heart can influence thyroid hormone levels. Furthermore, thyroid hormone can influence the heart’s response to stress such as in the case of ischemia. This bidirectional relationship highlights the need for a comprehensive understanding to optimize patient care. The articles published in this Research Topic on CV disease and thyroid function highlight further aspects of the complex relationship between the thyroid and the heart. Interestingly three of the published studies used Mendelian randomization (MR) as a new approach that employs genetic variations, mainly single-nucleotide polymorphisms (SNPs), and potential risk factors aiming to evaluate the potential causal relationship between them. This method is effective in reducing bias derived from confounding and reverse causation (1). Zhang et al. used MR to assess the relationship between TH and carotid intima-media thickness (CIMT) which is an early sign of atherosclerosis. The authors showed that lower levels of Free Thyroxine (FT4) within the normal range and hypothyroidism were associated with increased CIMT, whereas thyroxine therapy was associated with a reduction in CIMT. However, using multivariate MR, FT4 was not linked to CIMT, but only apolipoprotein (apo) A-I and B. Intriguingly, high FT4 levels were associated with high circulating apoA-I levels, and increased TSH was associated with high apoB levels. In the context of CV risk, Jin et al. confirmed, using the MR approach, the causal relationship between hypothyroidism and coronary heart disease. In the same study, this relation was partially mediated by HbA1c. These data are proof of the interplay of TH action with different CV risk factors potentially inducing atherosclerosis and thus increasing CV risk. In the context of heart failure, Liu et al., using MR, showed a causal relationship between hyperthyroidism and all-cause heart failure, suggesting a genetic link between the two conditions. This result further supports the pathophysiological role of TH in heart failure. Experimental studies have shown that altered TH metabolism may induce abnormal changes in cardiac structure and function that favor the progression of heart failure (2). Clinical studies have demonstrated the negative prognostic impact of the altered TH metabolism, even considering the early and middle abnormalities such as subclinical hypothyroidism, hyperthyroidism and low T3 syndrome (3). Moreover, TH treatment in an experimental context has been shown to exert cardioprotective effects through modulation of different key cellular pathways, including preservation of mitochondrial activity and morphology, antifibrotic and proangiogenic actions, and promotion of cell regeneration and growth after the postischemic injury (4). It is noteworthy that TH dysfunction can be considered as part of the hormonal and metabolic derangement in heart failure, which may induce the progression of the disease both at the level of the heart and other organs, making it a systemic disease (5).

With respect to left ventricular function, Li et al. showed that patients with overt hyperthyroidism present with diastolic dysfunction in only 30% of cases, and these patients have high circulating levels of connective tissue growth factor (CTGF) compared to hyperthyroid patients with normal diastolic function. Interestingly, CTGF is considered a type of pro-fibrotic factor and a new marker of cardiac dysfunction in patients with heart failure. Excessive CTGF can induce myocardial hypertrophy and also the proliferation of cardiac fibroblasts and deposition of extracellular matrix. This is a new result showing left ventricular diastolic dysfunction, as a sign of myocardial stiffness, in the presence of high CTGF levels, which is consistent with the evidence of the relationship between CTGF expression and the degree of fibrosis in papillary thyroid carcinoma. Finally, Shen et al. showed, in a retrospective study of patients with acute aortic dissection (AAD), that a one standard deviation increase in FT4 levels was associated with a 31,9% increased risk of MACE and a 36,1% of in-hospital mortality whereas a higher FT3/FT4 ratio was associated with a 20% reduction in MACE risk. Taken together, these results highlight the importance of assessing thyroid function in the context of patients with CV disease, both in terms of disease cure and cardiovascular prevention. The thyroid can be viewed as a conductor that oversees the operation of numerous organs and systems, such as the immune system, the inflammatory system, and hormonal pathways.

Changes in thyroid metabolism within the normal range could be viewed as homeostatic responses to preserve the functional balance of each organ and between organs. The demonstration of this fine and systemic tuning exerted by thyroid on the CV system is provided by the evidence that minimal alterations in thyroid metabolism can be detrimental to this system. These effects can be either direct, through morpho-functional alterations, as shown Li et al., and indirect by alterations in the known CV risk factors, such as glucose and lipid metabolism, as shown by Zhang Z and Jin. From the perspective of precision medicine, it is very important to further study the close relationship between the thyroid and the cardiovascular system in order to identify the key elements for the treatment of thyroid conditions, with a view to the prevention and treatment of cardiovascular disease.

## References

[1] Richmond RC Smith GD. Mendelian Randomization: concepts and scope. Clod Spring Harb Perspect Med. (2022) 12:a040501. doi: 10.1101/cshperspect.a040501 PMC8725623 34426474

[2] Pantos C Mourouzis I Cokkinos DV. Thyroid hormone and cardiac repair/regeneration: from Prometheus myth to reality? Can J Physiol Pharmacol. (2012) 90:977–87. doi: 10.1139/y2012-031 22762197

[3] Iervasi G Molinaro S Landi P Taddei MC Galli E Mariani F. Association between icreased mortality and mild thyroid dysfunction in cardiac patients. Arch Intern Med. (2007) 167:1526–32. doi: 10.1001/archinte.167.14.1526 17646607

[4] Mantzouratou P Malaxianaki E Cerullo D Lavecchia AM Pantos C Xinaris C. Thyroid hormone and heart failure: charting known pathways for cardiac repair/regeneration. Biomedicines. (2023) 11:975. doi: 10.3390/biomedicines11030975 36979954 PMC10046827

[5] Mastorci F Sabatino L Vassalle C Pingitore A. Cardioprotection and thyroid hormones in the clinical setting of heart failure. Front Endocrinol (Lausanne). (2020) 10:927. doi: 10.3389/fendo.2019.00927 32047475 PMC6997485

